# Human EEG and Recurrent Neural Networks Exhibit Common Temporal Dynamics During Speech Recognition

**DOI:** 10.3389/fnsys.2021.617605

**Published:** 2021-07-08

**Authors:** Saeedeh Hashemnia, Lukas Grasse, Shweta Soni, Matthew S. Tata

**Affiliations:** Canadian Centre for Behavioural Neuroscience, Department of Neuroscience, University of Lethbridge, Lethbridge, AB, Canada

**Keywords:** EEG, artificial neural network, speech tracking, auditory, theta, recurrent, RNN

## Abstract

Recent deep-learning artificial neural networks have shown remarkable success in recognizing natural human speech, however the reasons for their success are not entirely understood. Success of these methods might be because state-of-the-art networks use recurrent layers or dilated convolutional layers that enable the network to use a time-dependent feature space. The importance of time-dependent features in human cortical mechanisms of speech perception, measured by electroencephalography (EEG) and magnetoencephalography (MEG), have also been of particular recent interest. It is possible that recurrent neural networks (RNNs) achieve their success by emulating aspects of cortical dynamics, albeit through very different computational mechanisms. In that case, we should observe commonalities in the temporal dynamics of deep-learning models, particularly in recurrent layers, and brain electrical activity (EEG) during speech perception. We explored this prediction by presenting the same sentences to both human listeners and the Deep Speech RNN and considered the temporal dynamics of the EEG and RNN units for identical sentences. We tested whether the recently discovered phenomenon of envelope phase tracking in the human EEG is also evident in RNN hidden layers. We furthermore predicted that the clustering of dissimilarity between model representations of pairs of stimuli would be similar in both RNN and EEG dynamics. We found that the dynamics of both the recurrent layer of the network and human EEG signals exhibit envelope phase tracking with similar time lags. We also computed the representational distance matrices (RDMs) of brain and network responses to speech stimuli. The model RDMs became more similar to the brain RDM when going from early network layers to later ones, and eventually peaked at the recurrent layer. These results suggest that the Deep Speech RNN captures a representation of temporal features of speech in a manner similar to human brain.

## 1. Introduction

Biological systems have evolved highly sophisticated mechanisms for perceiving and interpreting sensory input. Since the introduction of the first perceptron networks by Rosenblatt (1958) in the middle of the previous century, researchers have sought to learn lessons from biological neural architectures to create artificial ones that can learn to perceive. Recent work has yielded dramatic advances of deep Artificial Neural Networks (dANNs) and Recurrent Neural Networks (RNNs) that accomplish visual and auditory perceptual tasks with near-human-like performance (Oord et al., 2016; Huang et al., 2017; Kell et al., 2018; Kubilius et al., 2018). However, developments of machine learning for applications (such as image classification, automatic speech recognition, etc.) have proceeded so rapidly that the companion domains of neuroscience (i.e., vision science, auditory neuroscience) have become largely uncoupled and empirically disconnected from the state-of-the-art in machine learning. This situation exposes a range of interesting questions: Do convolutional deep neural networks and recurrent networks emulate the hierarchical and feedback processes of the human brain? Do they even extract the same features from the sensory world? We think there is good reason to postulate that the answer is “yes” and proceed to probe the similarities and differences between biological and artificial systems. Here we report meaningful similarities between human brain electrical dynamics captured by electroencephalography (EEG) and the activation dynamics of units in the Mozilla Deep Speech RNN during a speech perception task.

Understanding the similarities between brain and artificial neural networks is promising for two reasons: First, ANNs might capture important computational principles that the brain has also evolved to implement. In that case, they might act as useful models of brain computations, albeit by very different computational mechanisms. Second, a deep understanding of the similarities and differences between biological and (very good) artificial networks might substantially lead to faster and less data-intensive ways to train biologically-inspired artificial networks. Although dANNs are quite good, they are trained quite differently from the human brain (no infant learns to perceive by watching 10,000 h of YouTube videos). Thus, there are compelling reasons to consider how biological and artificial systems might be convergent (and divergent) in how they extract sensory features, and map those onto outputs.

Recent authors have begun to work toward this objective, with the particular goal of developing benchmarks to measure similarities between biological and artificial networks. For example, brain-score (Schrimpf et al., 2018) consists of several neural and behavioral metrics in the context of visual object perception. These provide a measure of similarities between the internal representation of an artificial network, such as DenseNet-169 (Huang et al., 2017) or CorNet-S (Kubilius et al., 2018), and the responses of single neurons in specific visual cortical regions. Further behavioral metrics compared network and human task performance (Schrimpf et al., 2018). Another suggested benchmark is Representational Similarity Analysis (RSA) in which network latent representations are compared with the associated brain responses in order to examine similarities in how the biological and artificial systems differentiate between a set of stimuli (Nili et al., 2014). These measures have been applied to visual object recognition and have demonstrated some similarities, despite the vast differences in biological and machine computational mechanisms (Khaligh-Razavi and Kriegeskorte, 2014).

By contrast, the domain of auditory perception has been less investigated. Despite keen interest and remarkable progress in speech recognition networks within the field of machine learning, these sophisticated networks have not been well-compared with the human speech perception system. In a recent paper, Kell et al. (2018) trained a convolutional network that achieved human-like performance on music genre and speech classification tasks. The activity within hidden layers of that network predicted patterns of voxel activity in human auditory cortex during speech perception, as measured by functional Magnetic Resonance Imaging (fMRI). Furthermore, they observed a hierarchical organization of this similarity, with early network layers better explaining primary cortex signals and deeper layers related to extra-primary cortex. This work further supports the notion that deep ANNs could capture computational principles that are employed by the brain during auditory perception. However, since hearing is fundamentally spectrotemporal, the fast-changing dynamics within recurrent networks (RNNs) such as Mozilla's Deep Speech are also of particular interest. Such networks are quite good in extracting speech from time-varying sound (Hannun et al., 2014) and mapping spectrotemporal information onto text output. However, it is unclear if the underlying temporal mechanisms are similar to speech processing in the brain. Comparing such a network with the brain representation of speech could help to understand whether the network is a good fit to model characteristics of the auditory system of speech perception.

Recent advances in the neuroscience of speech and language provide useful metrics with which to compare biological and artificial speech perception systems. The mechanisms of speech perception that track linguistic events in a time-varying speech signal have been revealed in phase, amplitude, and spectral features of cortical dynamics as measured by EEG, magnetoencephalography (MEG), and electrocorticography (ECoG). For example, cortical delta band oscillations are synchronized with words and phrases (Ding et al., 2016; Kösem et al., 2016; Meyer et al., 2017). Importantly, alignment of delta oscillations to linguistic components of speech is learning-dependent: the phenomenon only occurs for speech in a familiar language. Similarly, theta-band signals track the acoustic amplitude envelope related to the ~5 Hz syllable rate of speech (Ghitza, 2012, 2013; Giraud and Poeppel, 2012). Successful comprehension of speech is shown to modulate this theta-tracking phenomenon, suggesting that it reflects learned mechanisms of speech perception. For example, theta-tracking is reduced in when speech was made unintelligible by various distortions of the acoustic signal such as time compression (Ahissar et al., 2001), by removing spectrotemporal features (Peelle et al., 2013; Ding et al., 2014), by distorting the envelope itself (Doelling et al., 2014), by adding noise (Luo and Poeppel, 2007; Vanthornhout et al., 2018), or by distorting phonological information (Mai et al., 2016). Nevertheless, it seems that the brain is able to track the amplitude envelope in the theta band even when the speech was in an unfamiliar language (ding; Soni and Tata, 2021), when the signal was obscured (Zoefel and VanRullen, 2016), when speech was time-reversed (Howard and Poeppel, 2010), or when phonemes were omitted while preserving the low-frequency envelope (Hambrook et al., 2018). Thus, theta-band tracking of the amplitude envelope seems to be modulated by, but not entirely dependent on a learned mechanism.

Since brain electrical dynamics seem to be important for cortical mechanisms of speech perception, we reasoned that a very good RNN trained for speech recognition should exhibit similar temporal dynamics of the activations of its hidden units, particularly in recurrent layers. In order to investigate if these temporal features are in common with human brain responses, we presented a set of identical speech stimuli to a group of human participants as well as to a trained Mozilla Deep Speech network. We recorded brain electrical activity of the subjects using electroencephalography and compared EEG dynamics to speech with the network internal representations of the same speech stimuli. Following the literature on theta-band (~4–8 Hz) EEG phase tracking of the speech envelope, we show that the temporal responses of a trained network also tracked the speech envelope (relative to an untrained network). We also used the previously established benchmark, RSA, to show that human brains and the Deep Speech network differentiate similar features of speech.

## 2. Methods

Human EEG data was recorded as part of a larger experiment to compare native and non-native English speakers with respect to brain dynamics. These comparisons are reported elsewhere (Soni and Tata, 2021). Here we analyzed those data with respect to the relationship between native speakers of English and trained and untrained Deep Speech networks.

### 2.1. Participants

A total of 15 native English speakers, in the age range of 19–30 years old (mean ± SD: 21.47 ± 3.02), participated in this study. All participants were Canadians and were recruited from an undergraduate course at the University of Lethbridge for course credit (3 male; 1 left-handed). All participants had normal or corrected-to-normal vision and reported no history of neurological or psychiatric disorders. The study was in accordance with the Declaration of Helsinki and approved by the Human Subjects Ethics Committee of the University of Lethbridge. The data from all 15 participants were analyzed in this study.

### 2.2. Stimuli

Twenty-five unique speech utterances were presented to participants. Each utterance was made of two unique consecutive sentences that were individually meaningful but not necessarily related. The length of each sentence was 3–4 s, resulting in a total length of 5.5–6.5 s for each stimulus. Each speech stimulus contained 12–21 words (16.24 ± 2.28), 19–31 syllables (24.28 ± 3.13), and 52–89 phonemes (70.20 ± 8.72) per stimulus and were presented four consecutive times for each participant in a random order.

Speech sentences were selected from the TIMIT Acoustic-Phonetic Continuous Speech Corpus (Garofolo et al., 1993). The corpus contains time-aligned orthographic, phonemic, and word transcriptions of over 600 speech samples of different dialects of American English. Speech stimuli chosen for this study were all spoken by male speakers from two dialect regions. They were sampled at 16 KHz and normalized to the root mean square (RMS) amplitude before presentation.

### 2.3. Task Procedure

An Apple Mac Pro with a firewire audio interface (M-Audio Firewire 410) was used as a presentation device and the presentation was fully automated using MATLAB programming space (The MathWorks Inc., Natick, MA, USA) and Psychophysics Toolbox functions (Brainard, 1997), which were running on Apple Computer's Core Audio Framework (Mac OS 10.6). An Electrical Geodesics Inc. Net Station data acquisition software was used to record EEG. All stimuli were presented in a free-field sound attenuated room where a studio-grade audio monitor (Mackie HR624 MK-2) was located on the auditory front midline of the participant with a 1 m distance. Participants used a keyboard on a table close to them to report behavioral responses.

A monitor was located in from of the participants and was used to display some task descriptions before the task started, during a break, and after the task. It also was displaying a “+” sign at the center to identify a place for the participants to focus their eyes while listening. The group of selected utterances were presented in a random order, but each were presented four times consecutively, resulting in a total of 100 trials with one self-paced break after 50 trials. After each trial, participants had 30 s to type what they heard as best they could using a keyboard in front of them and then press “Enter” to continue.

### 2.4. EEG Data and Preprocessing

During the experiment, EEG signals were recorded via an Ag/AgCl 128-electrodel net (Electrical Geodesics Inc., Eugene, OR, USA) at 500 Hz sampling rate. The net size was chosen to fit the participant's head and the electrode impedances were checked to remain below 100 *KΩ*. EEG signals were preprocessed after the recording with Brain Electrical Source Analysis (BESA; Megis Software 5.3, Grafelfing, Germany) by first band pass filtering between 0.5 and 30 Hz and then replacing bad channels with a spline interpolation of the neighboring channels. After that, eye blinks and movements were filtered out using spatial filters (Ille et al., 2002) and then the signals were re-referenced to an average reference. We used EEGLAB (Delorme and Makeig, 2004) and customized code in MATLAB (MATLAB version 9.1.0; The Mathworks Inc., 2016, Natick, MA, USA) for the rest of the analysis. Data was FIR filtered between 1 and 20 Hz and downsampled to 250 Hz for further analysis. Each trial was extracted starting 700 ms before and ending 7,800 ms after the onset of stimulus presentation.

### 2.5. DeepSpeech Network

**Figure 1:**
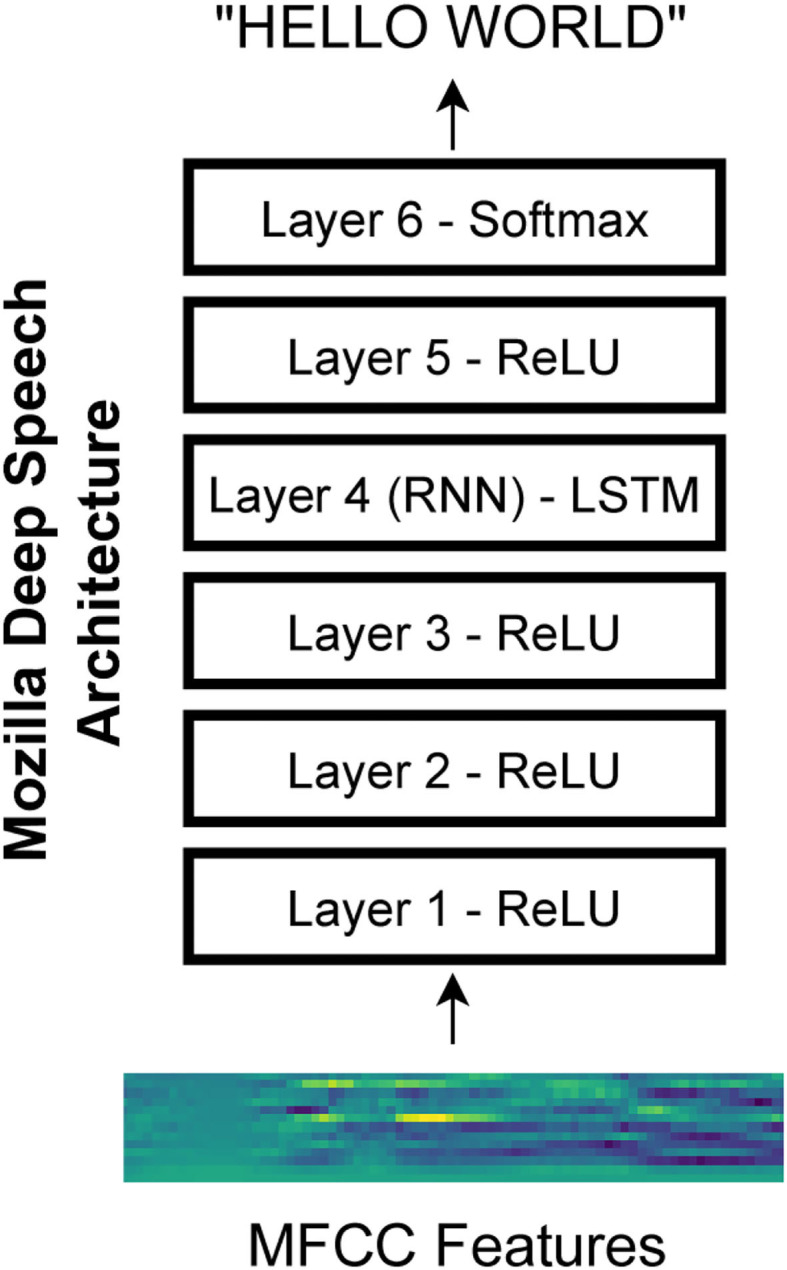
Mozilla deep speech architecture. The recurrent neural network contains five layers of hidden units where the first three layers as well as the fifth layer use a clipped rectified-linear activation function and the fourth layer is a recurrent layer. The last layer uses a softmax function to select the most probable alphabet letter at each timepoint (Hannun et al., 2014).

We used an open-source implementation of a speech recognition network called Deep Speech from Mozilla (https://github.com/mozilla/DeepSpeech). The network architecture is based on research from Baidu (Hannun et al., 2014). Specifically, we used version 0.6.1 of the network. The network architecture consists of three initial layers with clipped rectified-linear (ReLU) activation, an LSTM (i.e., long short-term memory) layer (Hochreiter and Schmidhuber, 1997), another layer with ReLU activation, and a final output layer with softmax activation (Figure 1). The Mozilla DeepSpeech implementation used in this paper has a unidirectional LSTM layer unlike the original DeepSpeech paper which used bidirectional LSTM layers. The network is trained using the Connectionist Temporal Classification (CTC) loss (Graves et al., 2006) to output alphabetical characters corresponding to a text transcript of the speech. This loss allows the network to learn to produce the correct output without requiring that the transcript be force-aligned to the audio.

### 2.6. Data Analysis

The acoustic envelope of each speech stimulus was first calculated by taking the absolute value of the Hilbert transform, then low-pass filtering at 25 Hz. We employed a least-square Finite Impulse Response (FIR) filter for this purpose. The filtered envelope was then downsampled to match the sampling rate of the temporal dynamics of the network output. Its first derivative was then calculated; then negative values were replaced by zero to make a positive half-wave rectified representation of the envelope. This was normalized to the summation of the amplitudes to make a standard area under the curve of 1. The resulting signals, which show acoustic modulations of stimuli, were used for subsequent cross-correlation between either the human EEG signals or the network activation signals.

EEG responses across the four repetitions of each stimulus were first averaged to reduce the noise. They were then averaged across 12 selected frontocentral channels (EGI sensors 4, 5, 6, 11, 12, 13, 20, 21, 25, 113, 119, 124). These channels were selected to be consistent with a frontal midline focus of signals volume conducted from auditory and adjacent cortex on the supratemporal plane. For each participant, the resulting signals were then down-sampled to the sampling rate of pre-processed acoustic envelope of the corresponding stimulus and then cross-correlated with the envelope. The correlation functions were then averaged across the participants to reach a grand-averaged cross-correlation function per stimulus. The acoustic envelope of each stimulus was likewise cross-correlated with its matching output of each layer of the trained Deep Speech network. The results were then averaged across those nodes higher than the 20th percentile of the correlation values, separately for each stimulus. The purpose of this step was to only keep the nodes that contributed most to the dynamics of the network.

As a measure of comparison, cross-correlation between the preprocessed envelope of each stimulus and the EEG or network responses to a randomly chosen non-matching stimulus was also calculated. This cross-correlation between non-corresponding time series provided a signal representing a null hypothesis. Peaks in the resulting cross-correlation closely resembled the well-known N1-P2 complex of the classic auditory Event-Related Potential (ERP) and were consistent with previous work using this approach (Hambrook et al., 2018). We chose peaks for statistical analysis by two-tailed paired-sample *t*-test at ~140 and ~220 ms based on a priori expectation from that previous work. Finally, the cross-correlation between each stimulus envelope and matching or random outputs was re-calculated for 100 shuffles of an untrained network, then averaged across the shuffles, and grand averaged across the stimuli. This cross-correlation with untrained networks allowed us to consider the less interesting hypothesis that temporal dynamics in the trained network might simply be “inherited” from the dynamics of the speech signal itself and passed-through the layers of the network.

The major goal of this study was to find if the Deep Speech model shares a common feature map with the human brain speech recognition system. Representational Similarity Analysis is a technique that has been employed to compare computational model representations of a group of inputs with the brain responses to the same stimuli (Nili et al., 2014). In the RSA framework, Representational Dissimilarity Matrices (RDMs) are first calculated for the brain and the model responses. RDM is a symmetric square matrix containing distances between all pairwise representations of the group of stimuli (Nili et al., 2014). The brain and model RDMs are then compared using Spearman rank correlation coefficient.

Using RSA toolbox and a Euclidean distance measure, we calculated RDMs for each of the first five layers of the trained network, as well as all 100 shuffled untrained networks for the group of 25 stimuli. Separately, the RDM for the brain responses of each of 15 participants was also calculated by first pooling the EEG data of the 12 selected EEG channels together. These RDMs were then averaged across the repetitions of each presentation. The group of 15 obtained brain RDMs were then correlated with each of the network RDMs. For the untrained network, the Spearman correlation coefficients were averaged across the 100 RDMs per layer per EEG participant. As a result, there were 15 correlation coefficients per layer for the trained network, and the same number for the untrained networks. For each network layer, these values were then statistically compared (by *t*-test) to test if the representation of the trained network was significantly more similar to the brain representations than the untrained networks. We next correlated RDMs of the trained and untrained networks with the average RDMs of EEG participants to find the general pattern across the layers.

In the next step, we extracted EEG components in 2 Hz bands to find the frequency bands where network RDM shares more or less similarity with the brain RDM. EEG signals were first filtered using FIR gaussian low/band pass filters. Then their RDMs were calculated and averaged across the repetitions per participants and then across the participants. Finally, obtained EEG RDM was compared with the RDMs of each network layer using spearman correlation coefficient.

In the next step, we extended RSA to all EEG electrodes in order to consider if different brain regions are more or less similar to different network layers. We reasoned that different patterns of scalp topography associated with different layers might reflect hierarchical processing of speech features. For each participant, we calculated the RDM of each EEG electrode (128 in total) and then found its pairwise correlations with RDMs of the layers of the trained and untrained networks. For the untrained network, the resultant Spearman correlation coefficients were averaged across the 100 shuffles per network layer per EEG electrode, resulting in 15 coefficients. Distribution of the correlation coefficients of the trained network were then statistically compared with the untrained network in each layer to find the electrodes showing significant similarity. For visualizing the scalp topography of these electrodes, significant correlation coefficients between each averaged EEG RDMs across the participants and each trained network layer were kept and the rest of the coefficients were set to zero.

## 3. Results

### 3.1. Recurrent Layer of Deep Speech Network Is Phase-Locked to Speech Envelope With a Similar Delay as EEG Signal

**Figure 2:**
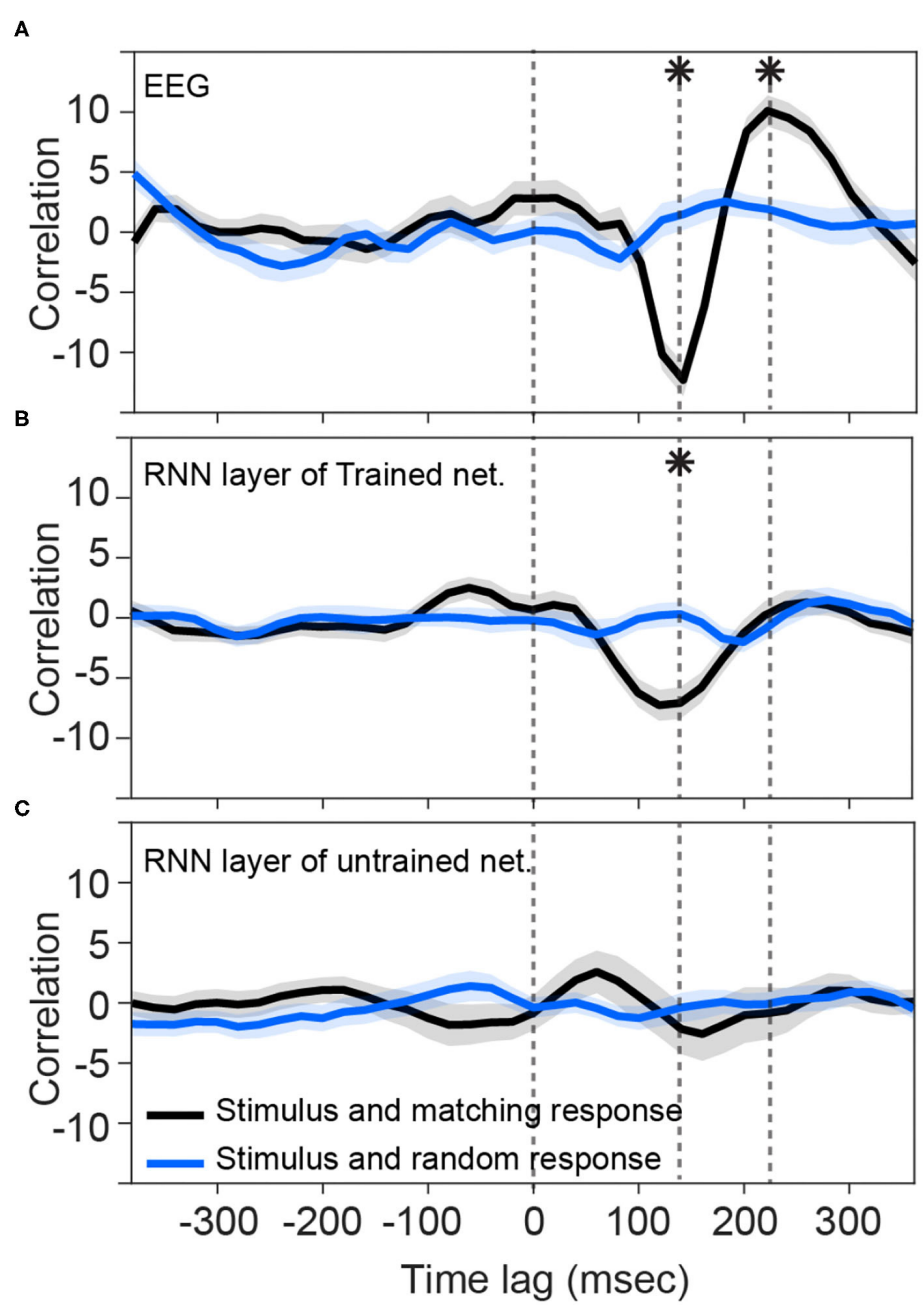
Tracking speech envelope modulations by EEG signals and recurrent output of the network. Correlation function between half-wave positively rectified derivation of the speech envelope and **(A)** average EEG signal across selected channels, **(B)** RNN output of the trained network averaged across the nodes in the highest 80 percentile, and **(C)** RNN output of each 100 shuffle untrained network averaged across the nodes in the highest 80 percentile are calculated. For **(C)**, correlation functions were then averaged across the shuffles. Solid lines indicate grand mean across the stimuli and their surrounding shadow indicates the Standard Error of Mean (SEM). Solid black curves indicate the correlation between matching pairs of stimuli and response signals, whereas the solid blue curves indicate non-matching pairs of stimulus-response that were chosen randomly. All the signals were time-aligned to the stimulus onset; thus, the positive peak lags represent the delayed responses to the stimulus and negative lags indicate possible brain/network predictions of the future input stimulus. Vertical dashed lines indicate time zero, and the time points of minimum and maximum correlations with EEG. Asterisks indicate where the matching and non-matching cross-correlations are significantly different at those timepoints (*t*-test, *p* < 0.05). Note that the value of the y-axis is the raw cross-correlation, not a correlation coefficient.

Consistent with previous studies (Hambrook et al., 2018), we found that cortical responses track acoustic envelope fluctuations with a slight delay (Luo and Poeppel, 2007). Figure 2A shows that cross correlation between the envelope of speech and EEG signals start to deviate from the baseline at about 100 ms after the presentation onset. It reaches to its minimum value (−12.3) at about 140 ms and then rises to reach the peak (10.1) at about 220 ms. These two peaks differ from the random stimulus-response significantly [*t*-test: min: $t_{\left(48\right)}=-7.4,p=17*10^{-10}$; max: $t_{\left(48\right)}=5.2,p=36*10^{-7}$]. Figure 2B shows that the trained network output of RNN layer also starts to track input fluctuations after about 80 ms. Although its deviation from the baseline is less than what we observe in EEG signal, its trough (−7.3) lines up with EEG in latency (~130–140 ms) and it is significantly different from the cross-correlation of non-matching stimulus-response pairs [*t*-test: $t_{\left(48\right)}=-4.5,p=48*10^{-6}$]. The later positive peak seen in the human EEG data seems absent, however, and is close to zero (0.16) and does not differ from the threshold [*t*-test: *t*_(48)_ = 0.79, *p* = 0.43]. Figure 2C, on the other hand, shows that the RNN layers of untrained networks are not phase-locked to the stimulus at those timepoints (*t*-test: *p* > 0.05).

### 3.2. Not All the Layers in the Trained Network Track Speech Modulations Similar to the Brain

**Figure 3:**
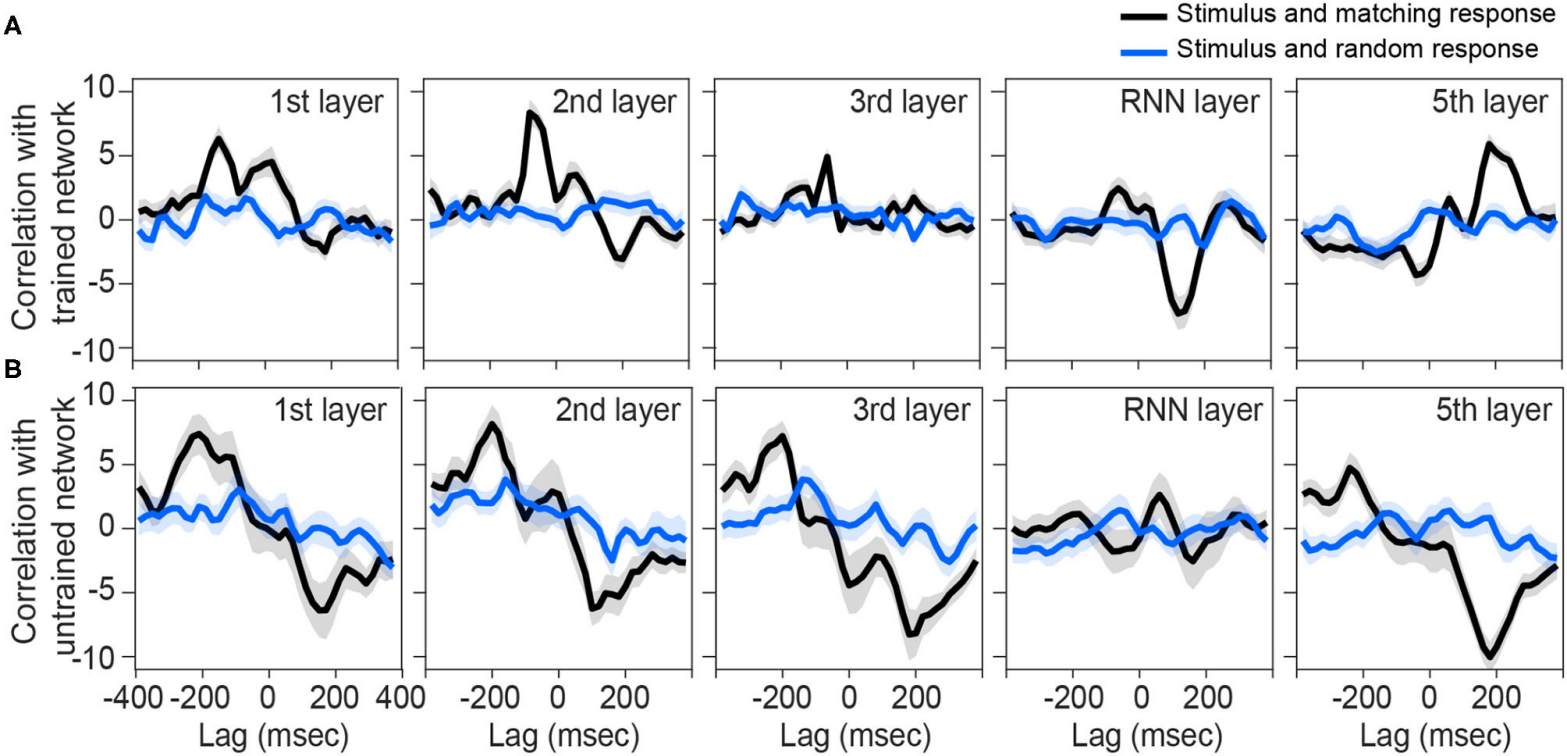
Correlation between preprocessed envelope of stimuli and the output of each layer. Correlation function between half-wave positively rectified deviation of the speech envelope and each layer output of the **(A)** trained network and **(B)** each 100 shuffle untrained network are calculated, then averaged across the nodes in the highest 80 percentile. For **(B)**, correlation functions were then averaged across the shuffles. Solid lines indicate grand mean across the stimuli and their surrounding shadow indicates the Standard Error of Mean (SEM). Black line indicates the correlation between matching pairs of stimuli-output signals, while blue lines indicate random pairs of stimulus and output. All the signals were time-aligned to the stimulus onset; thus, the positive lags represent the delayed responses to the stimulus and negative lags indicate possible network predictions of the future input stimulus.

We next sought to investigate other layers of the network to see if they also represent similar tracking patterns as the brain. Although the output of the first, second, and fifth layers are also correlated with their matching stimulus modulations (Figure 3A), the pattern is not near what we observed for the brain (Figure 2A). In other words, none of the rest of the layers is phase-locked to the input envelope at the same time-delay and correlation value as the brain is. Thus, the recurrent layer shows the most similar pattern to the human brain response to speech.

On the other hand, all layers of untrained networks (shown in Figure 2B) except the RNN layer, seem to represent some correlations with their matching stimulus on average, but with high variability. Although these networks are not trained to convert their speech inputs into text, they may still inherit and simply pass through input fluctuations which thus reflects in the correlation function. Similar reflections might have happened in other layers of the trained network.

### 3.3. Similarity Between Brain and Network Representations of the Stimuli Increases as the Input Travels to the Recurrent and Fifth Layer

We investigated other commonalities between the features encoded in the brain responses and those reflected by network outputs to the speech stimuli. One way to address this question is to find out if the brain and the network differentiate between pairs of stimuli in a similar way. For example, if the vector distance between the network outputs corresponding to two different speech streams is low, that means the network representations of these two speech streams are similar. Likewise, if the vector distance between EEG signals for two speech streams is low, it means that the brain represents these two stimuli similarly. Comparing these two sets of vector distances reveals whether patterns of similarity and dissimilarity are common across brain and artificial networks. Thus, the representational similarity analysis can be a measure of comparison between the model and the brain representations (Khaligh-Razavi and Kriegeskorte, 2014; Nili et al., 2014).

**Figure 4:**
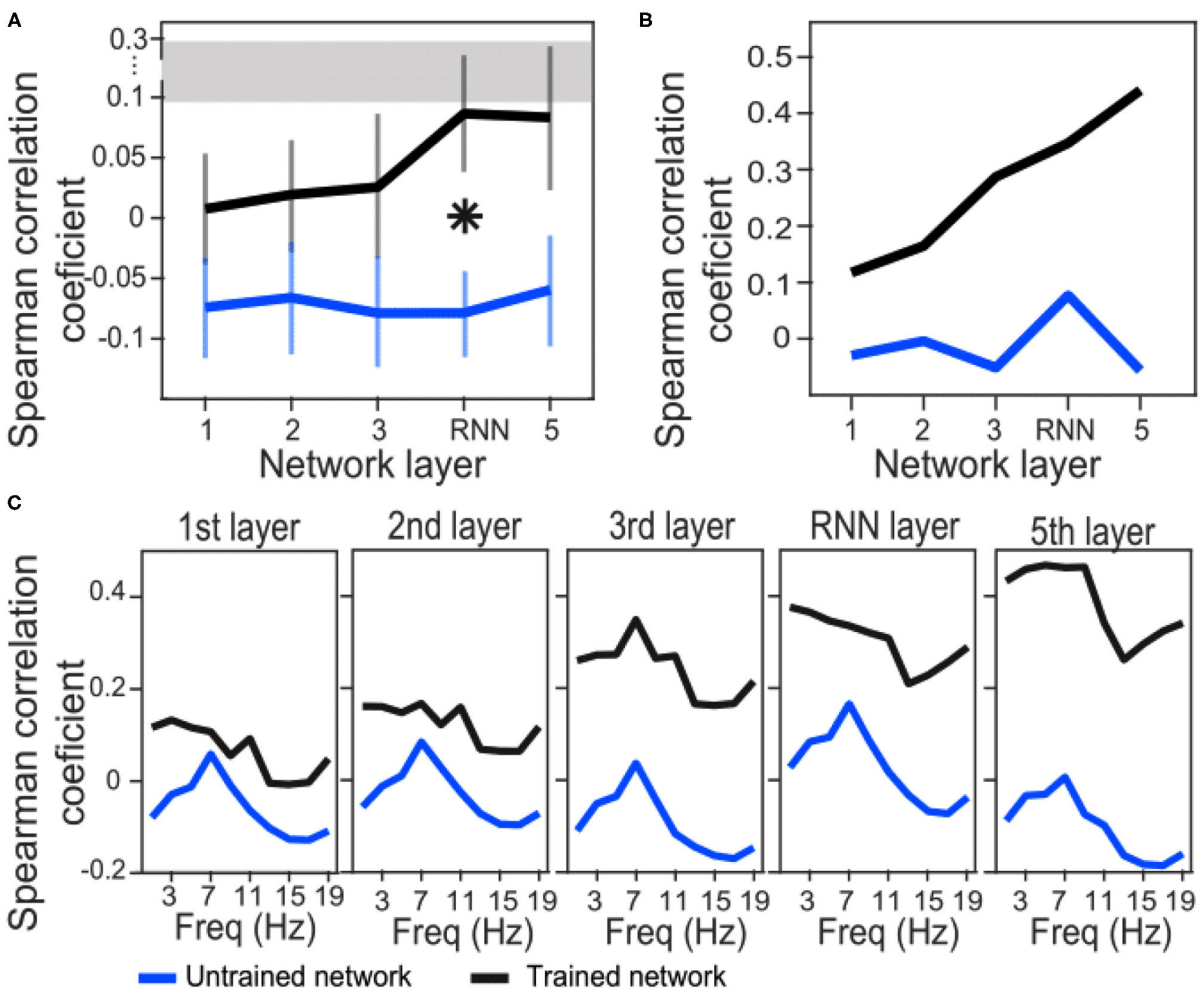
Correlation coefficients between brain and network RDMs. **(A)** Correlation coefficients between EEG RDMs of each participant and the trained/untrained network RDMs of each layer, averaged across the participants. For the untrained networks, correlation coefficients were first averaged across the 100 shuffles and then grand averaged across the participants. The error bars indicate SEM. Asterisk indicate statistically significant differences between trained and untrained networks in the specified layer (*t*-test, *p* < 0.05). The gray bar displays the noise ceiling with a lower bound of 0.094 and an upper bound of 0.295. **(B)** Correlation coefficients between averaged EEG RDMs across the participants and the trained/untrained network RDMs of each layer (only one untrained network is shown here for illustration). **(C)** EEG signals were first filtered in each 2Hz frequency bands and then RDMs were calculated. Correlation coefficients between averaged EEG RDMs across the participants and the trained/untrained network RDMs of each layer are plotted (only one untrained network is shown for illustration).

**Figure 5:**
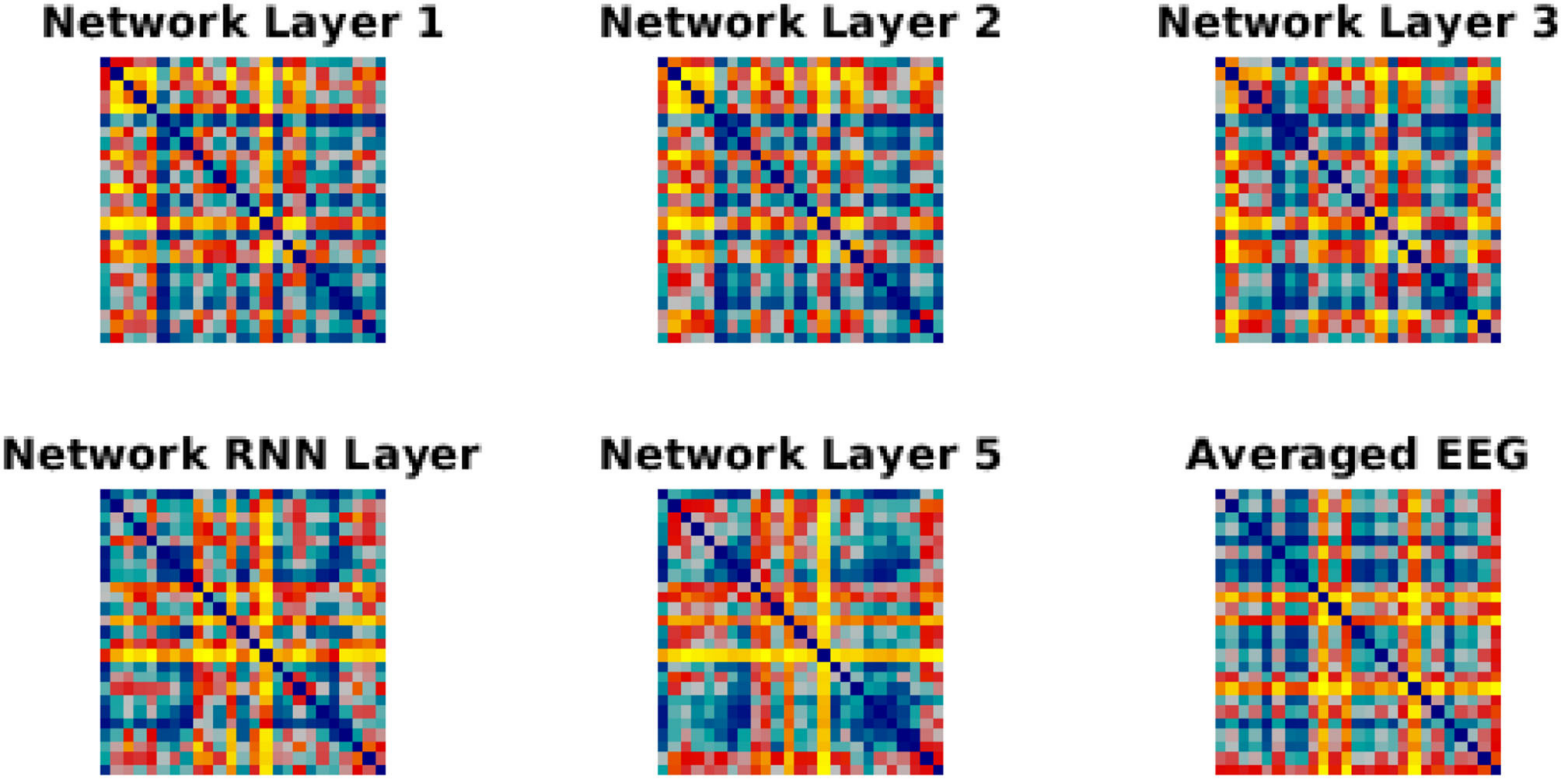
RDMs for network layer and EEG. Each similarity matrix was separately rank-transformed and scaled into the range [0, 1]. The EEG RDM is averaged across participants.

We have used Euclidean distance as a measure of differences between each pair of responses of either network or brain to make RDMs. The RDMs of EEG and each network layer of either trained or untrained networks were compared to each other with Spearman correlation coefficients. In contrast with the untrained networks, the averaged correlation values in the trained network across EEG participants showed an upward trend with a peak at the RNN layer (Figure 4A) which indicates that the representational dissimilarities of each layer of the trained network became more correlated to the representational dissimilarities of the brain when comparing to the previous layers. Also, comparing to untrained networks, the recurrent layer in the trained model was the only layer with an RDM that was significantly correlated with the EEG RDM [*t*-test, 1st layer: *t*_(28)_ = −1.3, *p* = 0.2; 2nd layer: *t*_(28)_ = −1.27, *p* = 0.21; 3rd layer: *t*_(28)_ = −1.38, *p* = 0.18; RNN layer: *t*_(28)_ = −2.73, *p* = 0.011; 5th layer: *t*_(28)_ = −1.87, *p* = 0.07]. Moreover, we observed that correlation between the trained network RDMs with the averaged RDMs of EEG participants increased as we progress through the layers (Figure 4B). Also, as an example of the untrained networks performance, one of them was randomly chosen and the correlations between its RDMs and the averaged RDMs of EEG participants are plotted. As anticipated by Figure 4A, compared to the trained network, these correlation values remained low. We have also included RDM plots for each network layer and the EEG averaged across participants in Figure 5.

In order to investigate whether the similarity between model and brain RDMs happen in a specific frequency band of the EEG signals, we next filtered EEG signals in 2 Hz narrow bands from 0 to 20 Hz and then repeated the RSA. Correlation coefficients of trained network drops at about 13 Hz in all the layers. However, a similar downward trend is also observed in all the layers of the untrained network at about 11–15 Hz. This might be a reflection of the inputs instead of the model features. On the other hand, a general trend from layer to layer is upward in the trained network indicating that the RNN and the last layers have the highest similarity with the brain representation of the stimuli.

By examining the similarity between EEG and network representations for each individual electrode we found regions across the scalp topography with significant correlations as compared to untrained networks. While about 40% of electrodes showed significant correlations with the RNN layer, only a few electrodes were correlated with the rest of the layers. Nevertheless, those correlations seemed to increase from each layer to the next.

## 4. Discussion

Early neural networks were inspired by ideas in neuroscience and psychology and were designed to be analogous to biological mechanisms. However, this is not always the case for recently developed networks, which are optimized for specific applications such as image classification or speech-to-text recognition. Thus, state-of-the-art dANNs may or may not share architectural and computational features with the brain systems and underlying mechanisms that perform related operations in humans (Khaligh-Razavi, 2014). However, these networks have shown great success in performing such tasks, approaching and in some cases exceeding, human performance. In that sense, recent successful dANNs are the closest computational models that we have for human-like perception. Mozilla's Deep Speech architecture can generate text transcriptions with good accuracy when given natural speech acoustic input. A key architectural difference of this (and related) network, relative to older speech recognition systems, is that it employs a recurrent layer. Given the substantial theoretical and empirical evidence for the importance of recurrence or “feedback” signals in cortical computations (e.g., Lamme and Roelfsema, 2000), we hypothesized that the temporal dynamics of recurrent layer activations in a trained Deep Speech network should exhibit some similarities with brain electrical dynamics during a speech listening task. We thus asked the question whether the internal representations of Deep Speech, either in early layers or the recurrent layer, parallel the brain representation of human speech features. We searched for commonalities between the two representations by comparing the latent outputs of the network with the recorded EEG signals of human brain.

We found that the recurrent layer in particular tracks envelope fluctuations of the speech signal in a similar way to the EEG signals measured at frontocentral electrodes. This was not observed in other layers of the trained network, nor in any layer of the untrained networks. On the other hand, the peaks in similarity at the theta band (~7 Hz) in untrained networks (Figure 4C), suggest that narrow-band envelope modulations are passed through the network regardless of training. This aligns with the observation that even incomprehensible speech is tracked at the theta band in EEG, at least to some degree (Howard and Poeppel, 2010; Ding et al., 2016; Zoefel and VanRullen, 2016; Hambrook et al., 2018; Soni and Tata, 2021). This finding suggests that envelope tracking by the auditory system is a phenomenon not limited to speech mechanisms *per se*, but rather related to more basic coupling between input dynamics and cortical responses. Learning speech, whether by a human brain or by a network, might refine the temporal fidelity with which these responses occur across frequency bands, leading to cross-frequency coupling that manifests as stronger cross-correlations between stimulus and response envelopes, such as shown in Figure 2.

Although this research has found that the recurrent layer tracks the envelope fluctuations of speech signals similarly to EEG, there is a potential confound due to the network architecture. Specifically, it is impossible to determine from the current research if the envelope tracking is due to the recurrent nature of the LSTM layer or if it is due to the position of the layer near the top of the network. This confound could be tested in future experiments that compare various network architectures that only use convolutional layers, including other state-of-the-art networks (Han et al., 2020; Zhang et al., 2020). Another valuable experiment could be to compare the early network layers to neural activity in the early auditory system, although there would be a difficult technical challenge in recording such neural activity in humans. Testing the representations learned by unsupervised networks such as variational autoencoders (Kingma and Welling, 2013) would be an interesting future area to explore. It is also important to note that the brain does not directly perform the speech-to-text task that the network is trained to perform.

Using Representational Similarity Analysis, our results also showed that similarity between the RDM of frontocentral EEG and network RDMs increased by progressing through early to intermediate layers of the trained network, from the first layer to the fifth one. However, only at the RNN layer was this similarity significantly different from the untrained networks. Moreover, when comparing the RDMs of EEG signals within frequency bands with the RDMs of network layers, the similarity was enhanced in almost all the frequencies as the layer number increased. Notably, this progression did not occur for untrained networks (Figure 4C), which suggests that it is a result of computations learned by the network for performing speech recognition.

### 4.1. Network Layer Matters

This study demonstrated that the Deep Speech network captures some spectrotemporal features in the speech stimulus in a manner similar to what the brain captures. However, this similarity is not the same across network layers. In fact, the RNN layer showed the most robust similarity of tracking structure compared to the brain signals (Figures 2, 3). This suggests that the recurrent layer performs computations on, or at least represents, time-varying features that are also represented by the neural processes underlying the scalp-recorded EEG at the frontal midline. In this sense, the RNN layer might model a spectrotemporal operation that is learned by the auditory cortex during language acquisition. This operation is presumably more elaborate than simply tracking the occurrence of amplitude modulations related to syllable stress, because even untrained networks seem capable of passing through this signal in the theta band (note in Figure 4C the presence of a peak at around 7 Hz across layers of the untrained network). One possibility is that the fluctuations of activity at more than one frequency are nested and aligned with the syllable rate, but only in the trained network (and the brain).

Another fascinating aspect of the RNN layer dynamics is the similar ~140 ms delay that also appears in the EEG. Traditionally the lag in EEG is interpreted as being due to transmission delay between peripheral auditory circuits and the cortex, and as such it does not seem to apply for artificial networks. Yet we see in the RNN layer the same peak at the same latency. One possibility is that this is how gating works in LSTM units, and the units are exhibiting this sensitivity to the temporal dynamics of prior information in the speech stream.

### 4.2. Network Layers and the Hierarchy in the Biological System of Speech Perception

The interesting upward trend in the representational similarity between the EEG RDM and network layers suggests hierarchical organization in the processing of speech features. In other words, the early layers, which do not seem to have a lot in common with the recorded activity of frontocentral electrodes, may fit other regions of the ascending auditory pathway related to early and simple processing of the sound stimuli. In this view, the recurrent layer processes later and more complicated stages of the speech recognition. This is consistent with previous results of an auditory cortex model (Kell et al., 2018) derived from a dANN trained to perform speech and music classifications. Kell et al. showed that a dANN consisting of 12 layers of feedforward (early layers) and fully connected (deep layers) performed similar to humans on word recognition and music genres classification. Activity in that network was capable of explaining human auditory cortical responses to sound stimuli measured by fMRI. Importantly, the authors found that the hierarchical structure of their dANN was reflected in the functional anatomy of the auditory system: the earlier layers of their network predicted primary or “core” auditory cortex activity whereas the intermediate and deep layers predicted signals in non-core auditory cortex voxels. This predictive performance dropped significantly for the fully connected layers, possibly because the function of those layers correlated with regions outside the auditory cortex (Kell et al., 2018). Our results are broadly consistent with such a hierarchy as suggested by our RDM analysis: we found that the correlation between early network layers and EEG was evident at only a few electrodes whereas the correlations with the RNN layer was disributed across a large network of EEG electrodes over temporal, central, and frontal regions. It is important to note that even though both the RNN layer and the EEG results use a similar representation, this does not mean that the mechanisms they use are necessarily the same.

To summarize, we found interesting similarities between brain electrical dynamics during speech perception and the time-varying activations of units in a trained recurrent neural network performing the same speech recognition task. Those similarities were consistent with a hierarchical arrangement of the representation of speech features. This result explored with EEG, considered along with related work with visual (Schrimpf et al., 2018) and auditory (Kell et al., 2018) functional MRI, suggests a compelling reason to further consider deep and recurrent neural networks as models of brain functions, despite the profound differences between the low-level computational mechanisms (i.e., biological vs. digital). Further investigations of the similarities and differences between such networks and the human brain under similar or identical perceptual tasks will advance development of ANNs and perhaps provide insights into brain computational mechanisms.

## Data Availability Statement

The raw data supporting the conclusions of this article will be made available by the authors, without undue reservation.

## Ethics Statement

The studies involving human participants were reviewed and approved by Human Subjects Ethics Committee of the University of Lethbridge. The patients/participants provided their written informed consent to participate in this study.

## Author Contributions

SH: conceptualization, formal analysis, visualization and methodology, analyzed data, wrote original draft of the manuscript and produced figures, and interpreted the results, all Network layers and the hierarchy in the biological system of speech perception under supervision of MT. LG: conceptualization, selected the RNN, trained and untrained the network, ran the stimuli through the networks, contributed in writing, edited and/or commented on manuscript, and interpreted the results, all under supervision of MT. SS: conducting the experiment, data curation, data collection and pre-processing, edited and/or commented on manuscript, all under supervision of MT. MT: conceptualization, supervision, visualization, methodology, extensively edited and/or commented on manuscript, project administration, supervised the experiment by SS as well as the analysis by SH and LG, interpreted the results. All authors contributed to the article and approved the submitted version.

## Conflict of Interest

The authors declare that the research was conducted in the absence of any commercial or financial relationships that could be construed as a potential conflict of interest.
